# Age-differentiated comparison of health-related quality of life and impacting factors in patients with COPD receiving long-term home non-invasive ventilation

**DOI:** 10.1186/s12890-025-03737-3

**Published:** 2025-06-07

**Authors:** Maximilian Zimmermann, Franziska Vocht, Doreen Kroppen, Daniel S. Majorski, Melanie P. Berger, Sarah B. Stanzel, Johannes F. Holle, Falk Schumacher, Tim Matthes, Wolfram Windisch, Maximilian Wollsching-Strobel

**Affiliations:** 1 Cologne Merheim Hospital, Department of Pneumology, Witten/Herdecke University, Kliniken der Stadt Köln gGmbH, Cologne, Germany; 2 Cologne Merheim Hospital, Department of Neurology, Kliniken der Stadt Köln gGmbH, Cologne, Germany; 3 Department of Rheumatology, Krankenhaus Porz am Rhein, Cologne, Germany; 4 Witten/Herdecke University, Cologne, Germany; 5 Institute for Medical Statistics, University Medical Center Goettingen, Göttingen, Germany

**Keywords:** COPD, Non-invasive ventilation, Health-related quality of life, Elderly patients

## Abstract

**Background:**

Non-invasive ventilation (NIV) is a well-established treatment for chronic hypercapnic respiratory failure (CHRF). While studies have demonstrated benefits for mortality, hospitalization rates, and health related quality of life (HRQL), evidence is particularly sparse regarding HRQL determinants in the older population.

**Methods:**

In a prospective, monocentric observational study, HRQL was assessed using the established Severe Respiratory Insufficiency Questionnaire (SRI). The study was prospectively registered in the German Clinical Trials Register on 17 June 2015 under the registration number DRKS00008759. Patients were categorized into two age-based groups: older patients (≥ 65 years) and younger patients (< 65 years). Multiple linear regression analyses were used to analyze factors on HRQL, including SRI scores, anemia, autonomy impairment, exacerbation history and other factors.

**Results:**

237 Patients with COPD with CHRF receiving NIV therapy were included. The mean SRI summary score was 49.9 ± 16.8. with 23.2% (*N* = 55) suffering from anemia and 36.7% (*N* = 87) experiencing *≥* 2 exacerbations annually. Autonomy impairment was observed in 49.4% (*N* = 117) of patients. The updated Charlson Comorbidity Index (uCCI) was 2.2 ± 1.86. No significant differences were found in SRI Summary Scale scores between age groups (*p* = 0.581), but notable disparities were present in the uCCI (*p* = 0.014). Multiple regression analysis revealed a negative association of exacerbation history (Young group: -9.2; 95% CI = -14.8/ -3.55 vs. Older group: -6.17; 95% CI = -11.91/ -0.43) and level of autonomy impairment (e.g. Level of Care 2 Young group: -13.91; 95% CI = -21.4/ -6.43 vs. Older group: -14.94; 95% CI = -22.64/ -7.24) on SRI scores with age-related differences. Anemia only had a negative association on the SRI scores in younger patients with COPD (Young group: -7.9; 95% CI = -14.0/ -1.75 vs. Older group: -1.78; 95% CI = -9.21/ 5.65).

**Discussion:**

Frequent exacerbations and a higher level of autonomy impairment had a negative association on HRQL across all ages. However only higher levels of impairment (≥ 2) have a detrimental impact on older patients. Anemia was a negative HRQL factor in younger patients, where it was more prevalent. Overall, HRQL was found to be comparably favorable in both older and younger patients, despite age-specific differences in influencing factors.

**Registration of the clinical trial:**

The study from which the data were analyzed was prospectively registered in the German Clinical Trials Register (DRKS00008759) on June 17, 2015.

**Supplementary Information:**

The online version contains supplementary material available at 10.1186/s12890-025-03737-3.

## Introduction

Chronic obstructive pulmonary disease (COPD) is one of the leading global health burdens, with more than 3 million deaths in 2019 and a steadily increasing prevalence [1]. With demographic changes and an ageing population, the number of patients with COPD who develop chronic hypercapnic respiratory failure (CHRF) is also increasing [2, 3].

Patients with CHRF experience a marked reduction in health-related quality of life (HRQL), which is increasingly recognized as a central outcome parameter in both clinical care and research [4]. The Severe Respiratory Insufficiency (SRI) questionnaire is a validated and disease-specific instrument designed to capture the complex aspects of HRQL in patients with CHRF [5-7]. In patients with COPD receiving non-invasive ventilation (NIV), SRI scores typically average around 50 out of 100, and the tool is sensitive to both improvements and deteriorations in HRQL [5, 8]. Several studies have defined the minimal clinically important difference (MCID) for the SRI, which is around *±* 5–6 points in the SRI summary score [9, 10]. NIV, when provided according to current guidelines, has been shown to improve HRQL in this population [11-14]. However, individual responses to NIV vary considerably, and several studies have identified factors that influence HRQL in patients with COPD and CHRF, including anemia, exacerbation frequency, and functional impairment as reflected by the level of care required [15-17]. These health-related factors are potentially modifiable and therefore relevant therapeutic targets. In contrast, age is a non-modifiable but clinically important factor. Although age itself is not a contraindication to NIV, and previous research suggests that NIV can improve HRQL in all age groups, it remains unclear whether the determinants of HRQL differ between younger and older patients [11, 18].

Given that older patients with COPD often have a higher burden of comorbidities, reduced autonomy and limited life expectancy, it is plausible that the relative influence of individual factors on HRQL may vary with age [3, 19-22]. A better understanding of age-specific HRQL determinants is essential to provide individualised and resource-conscious treatment strategies, especially considering increasingly limited healthcare resources and the growing importance of patient-centred outcomes [23, 24].

Therefore, the aim of this study was to investigate differences in HRQL between younger and older patients with COPD and CHRF receiving long-term NIV, with a focus on identifying age-specific determinants of HRQL. The results are intended to support a more personalised approach to the management of patients with COPD of any age on long-term NIV and provide clinically relevant factors for future research and therapeutic decision-making.

## Methods

The study was conducted as a prospective, monocentric, observational cohort study at the Department of Pneumology, Cologne-Merheim Hospital, Witten/Herdecke University, Cologne, Germany. The Ethics Committee for Human Studies, University of Witten/Herdecke, Witten, Germany, approved the study protocol for data collection [Ethical approval number: 68/2015]. Data collection was performed according to the ethical standards set out in the Declaration of Helsinki (last revised: 2013) [25]. Written consent was obtained. The study from which the data were analysed was prospectively registered in the German Clinical Trials Register on 17 June 2015 under the registration number DRKS00008759 [26]. The STROBE statements for cohort studies were followed when presenting the results of this study [27].

### Data collection and patient characteristics

Data were collected between June 2015 and October 2021. Study eligibility was assessed for all patients allocated to long-term home NIV for more than one month. For the final age-differentiated analyses patients with COPD with CHRF receiving NIV therapy were enrolled. Patient status was documented as inpatient or outpatient as described in previous publications investigating patients with COPD suffering from CHRF [15, 16].

Two groups were created according to the definition of the German Society for Geriatrics, which defines geriatric patients as individuals aged 65 years and older:< 65 years of age and patients ≥ 65 years of age [28].

Sex, age, body mass index (BMI), smoking status and pack years, living situation, indication for NIV, duration of NIV, if patients had participated in a pulmonary rehabilitation, diagnosis of depression and anemia defined according to the Word Health Organizations definition for females (Hemoglobin (Hb) < 12 g/dl) and males (Hb < 13 g/dl) were recorded [29]. Exacerbation frequency in the year before study inclusion was documented and dichotomized as ≤ 1 or ≥ 2 exacerbation(s) according to GOLD criteria [30]. The updated Charlson comorbidity index (uCCI) was used to identify potentially life-shortening comorbidities. It was used as a predictor for mortality within the year after data acquisition [31].

The level of autonomy impairment was documented as externally evaluated by the German Health Insurance Medical Service (Medizinischer Dienst der Krankenversicherung) according to standard criteria [32]. Here, five impairment levels (levels of care) are established: minor impairments (level 1), significant impairments (level 2), serious impairments (level 3), most serious impairments (level 4), most severe impairments to independence or ability that are associated with significant challenges for nursing care (level 5). Patient compliance was determined by reading the ventilator’s built-in software. Oxygen supplement levels for long-term oxygen therapy were documented as those used by the patient at the time of enrolment. HRQL was assessed using the SRI, developed, and validated specifically for patients suffering from chronic respiratory insufficiency [6, 33-35]. The SRI contains 49 items with seven subscales measuring different aspects of HRQL (respiratory symptoms, physical functioning, accompanying symptoms and sleep, social relationships, anxiety, psychological well-being, social functioning). The subscales can be aggregated into a summary scale. Each scale produces a score (0-100), with higher scores indicating a higher HRQL. All data were recorded on a standardized case report form (CRF), pseudonymized and electronically archived.

All patients received high-intensity NIV therapy with the aim of achieving maximum individual PaCO₂. Blood gas values were obtained at the time of enrolment, when long-term NIV therapy was already established. Due to the observational design of the study with no follow-up, systematic long-term adherence data were not collected.

Inclusion criteria were home NIV therapy for at least one month in COPD patients with CHRF aged ≥ 18 years. Exclusion criteria were lack of written informed consent and inability to speak and/or read German.

### Statistical analysis

The study was planned as a pilot study and a sample-size estimation was performed. The number of variables in the regression analysis has been determined considering that there should be a minimum of 10 observations for each variable as performed in previous studies. The data are presented in a descriptive manner as mean ± standard deviation (SD), unless otherwise stated. Groups were compared using statistical tests selected based on the distribution pattern and variance of the data. For metric characteristics with normal distribution and similar variances, Student’s t-test was used. In cases of normal distribution with different levels of variance, Welch’s test was used. For non-normally distributed characteristics with similar variances, the Mann-Whitney U test, and for non-normally distributed data with different variances, the Yuens test was performed. Ordinal characteristics were compared using the chi-squared test (Chi-X^2^). A *p*-value of *≤* 0,05 was considered statistically significant.

**Fig. 1:**
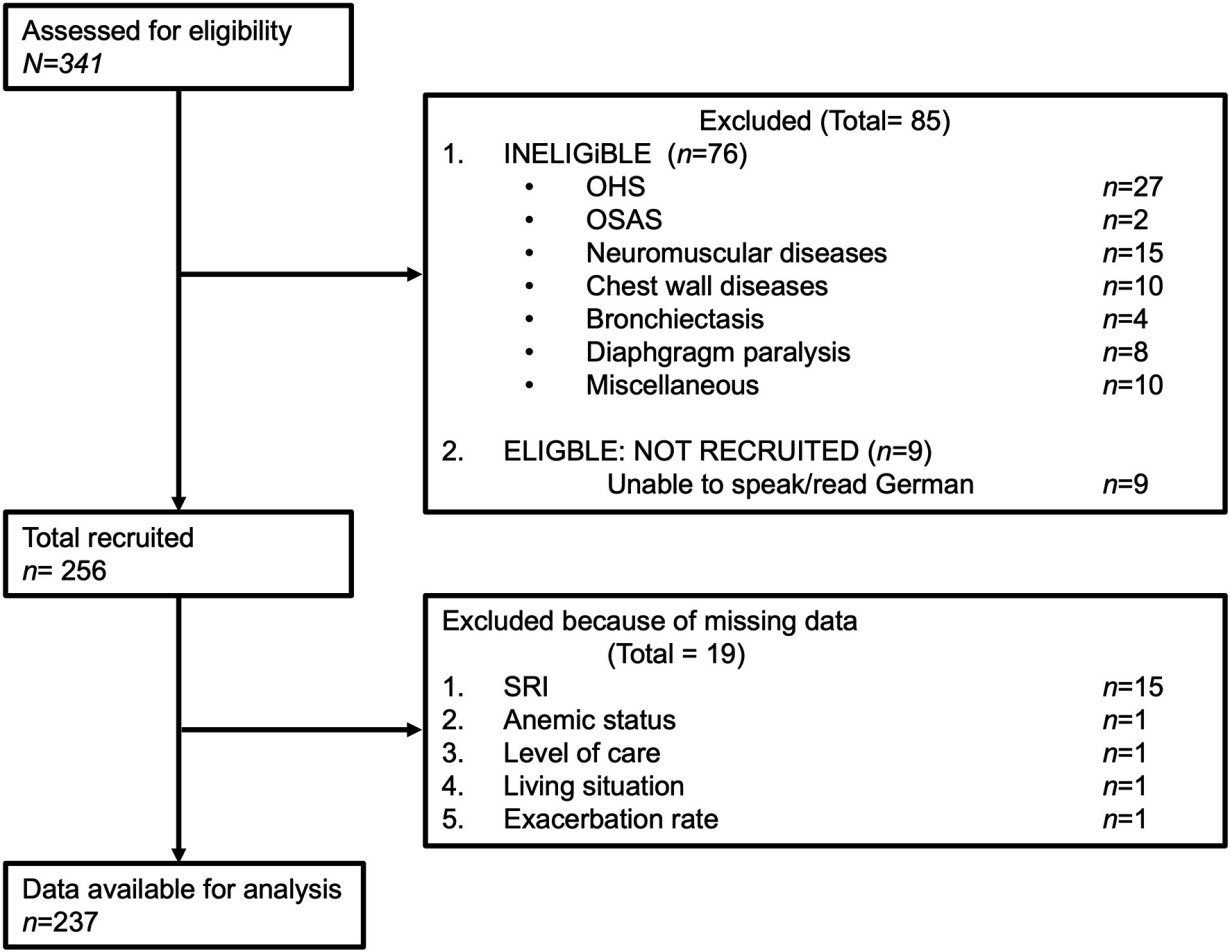
Flow diagram of subject recruitment and data availability. Abbreviation: N, number: OSAS, obstructive sleep apnea syndrome; OHS, obesity hypoventilation syndrome, NMD, neuromuscular disease; SRI, Severe Respiratory Insufficiency Questionnaire

To account for possible independent confounders on HRQL, multiple linear regression analyses were performed for the whole population of patients with CHRF receiving NIV. The following factors were integrated into the analysis: Age, sex, smoking status, pack years, BMI, duration of NIV, living situation, level of autonomy impairment (level of care 1–5), previous pulmonary rehabilitation, uCCI, exacerbation frequency, anemia and comorbid depression. After identification of the main confounders (level of autonomy impairment, anemia and exacerbation frequency), the following multiple linear regression analyses were performed for the age groups: Young and older patients with COPD suffering from CHRF patients receiving NIV. SRI scale scores were used as dependent variables to detect effects on HRQL in all analyses. SPSS V.26 (IBM SPSS Statistics for Windows, 2017) was used for the multiple regression analyses on a complete-case basis. Jamovi (Version 2.3.21.0) was used for descriptive statistics. Information regarding missing data and reasons for excluding participants are shown in Fig. 1. It was hypothesized that influencing factors on HRQL of life differed between age groups.

## Results

**Fig. 2:**
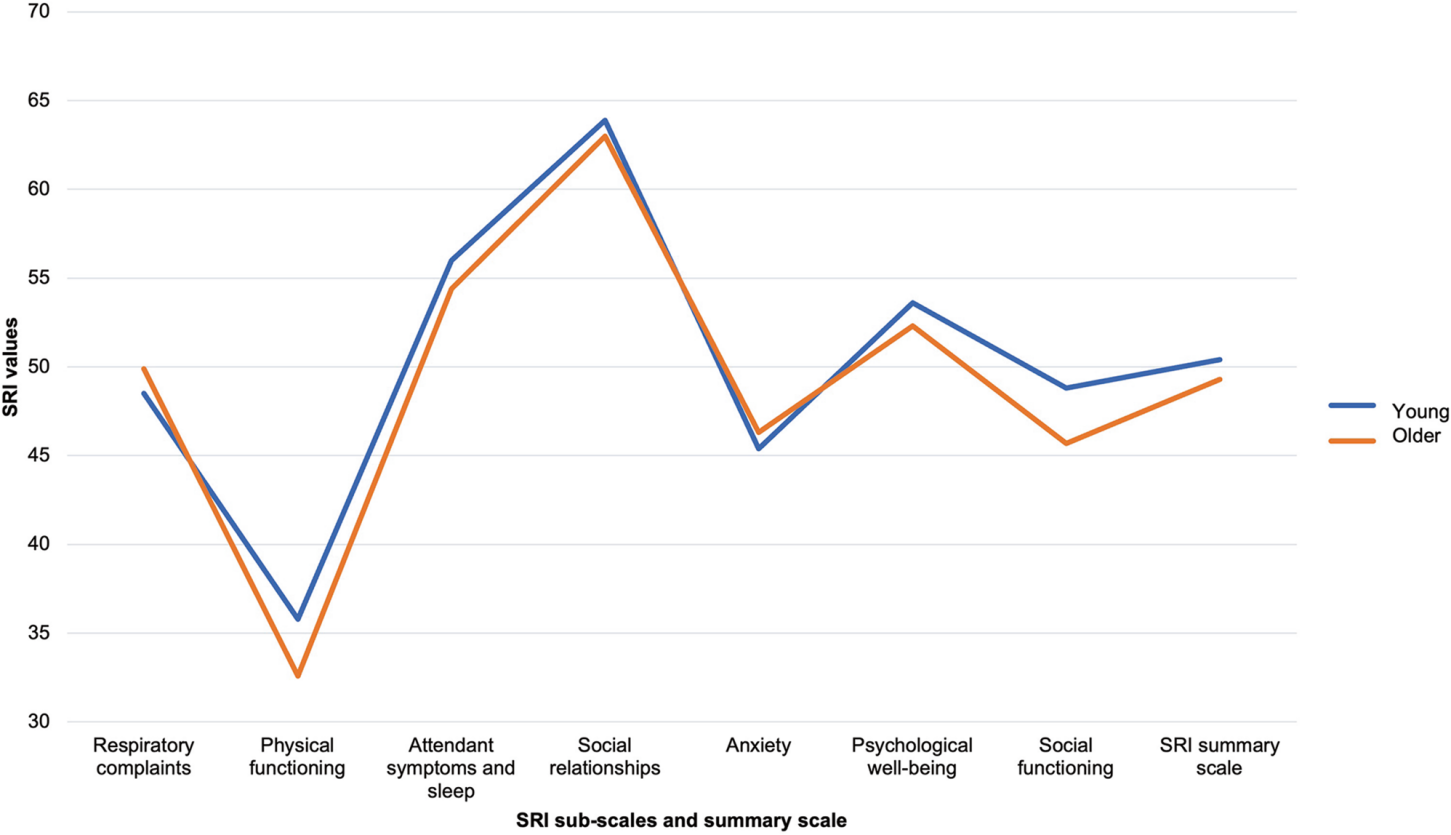
Comparison of SRI scores in young and older patients with COPD with type 2 CRF on NIV. Abbreviation: COPD, chronic obstructive pulmonary disease; CRF, chronic respiratory failure; NIV, non-invasive ventilation; SRI, Severe Respiratory Insufficiency Questionnaire

**Fig. 3:**
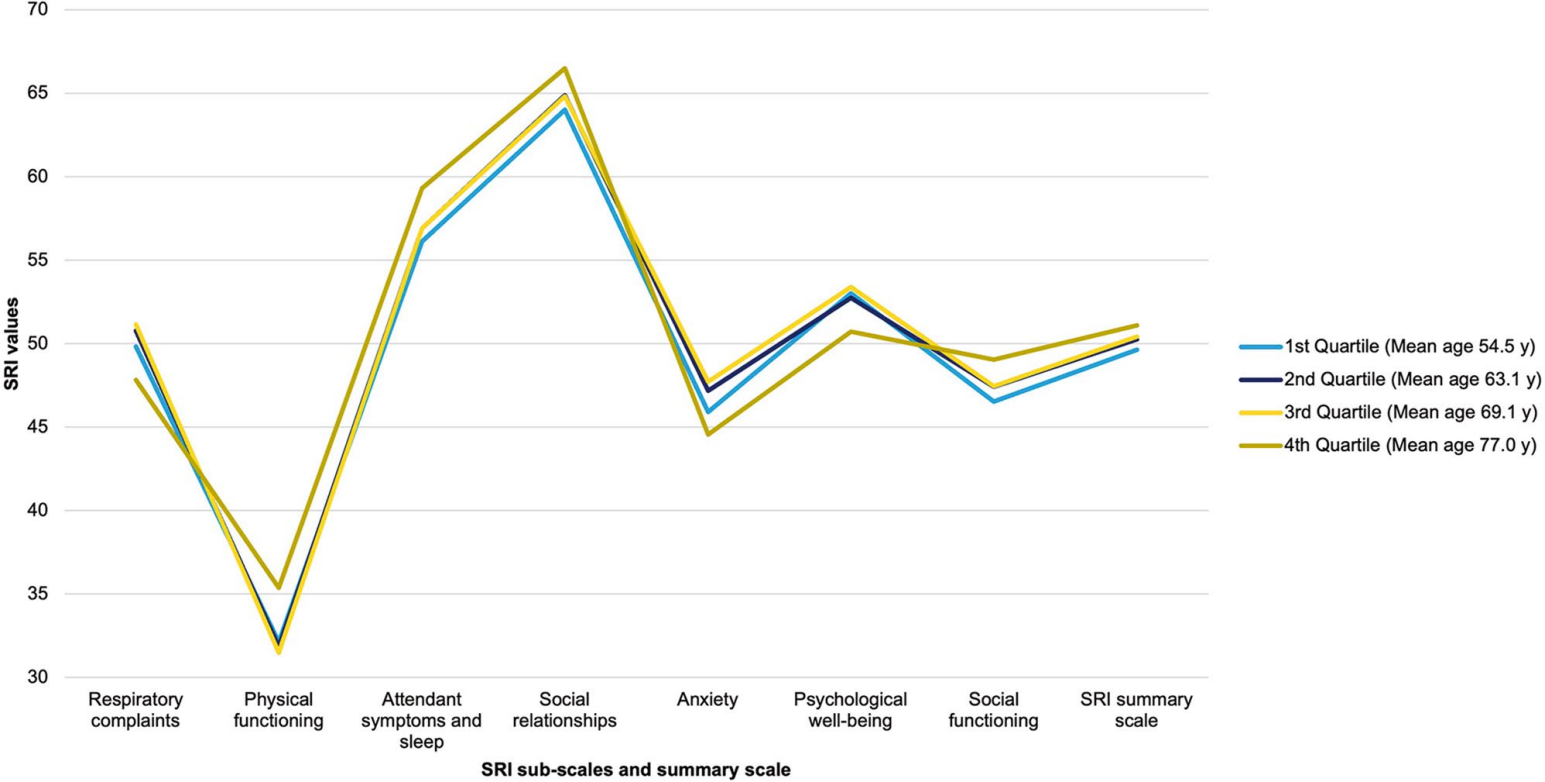
Comparison of Severe Respiratory Insufficiency Questionnaire (SRI) scores according to age. Legend: All patients with COPD with type 2 chronic respiratory failure allocated to non-invasive ventilation in the study were stratified into age quartiles for ease of visualization. Abbreviation: COPD, chronic obstructive pulmonary disease

A total of 341 patients were screened and 237 patients with COPD with CHRF and NIV therapy were included in the age-differentiated analysis. 41.8% (*N* = 99) of patients were enrolled for inpatient NIV control and 58.2% (*N* = 138) of patients were enrolled for outpatient NIV control. The mean SRI summary score was 49.9 ± 16.8. 23.2% (*N* = 55) patients suffered from anemia, 36.7% (*N* = 87) of patients had *≥* 2 exacerbations in the year before study inclusion. An impairment level was assigned to 49.4% (*N* = 117) of patients. The uCCI was 2.2 ± 1.86. Patient characteristics of both groups are described in Table 1. The uCCI was significantly higher in the older group than in the younger group (2.45 ± 1.93 vs. 1.93 ± 1.76; *p* = 0.014). The proportion of patients receiving LTOT was significantly higher in older patients (3.46 *±* 2.6 vs. 2.50 *±* 2.41; *p* = 0.021). Anemia occurred significantly more often in the younger age group (29.1% (*N* = 34) vs. 17.5% (*N* = 21) *p* = 0.045). The distribution of levels of autonomy impairment did not differ significantly between the two groups, although five patients in the older group and none in the younger age group were classified in care level four. No patients were classified as level five (Table 1). The primary unadjusted comparison of absolute SRI summary and subscale scores between the two age groups showed no statistically significant differences (Fig. 2). Figure 3 shows the SRI summary score and subscales for all patients per quartile.

**Table 1:**
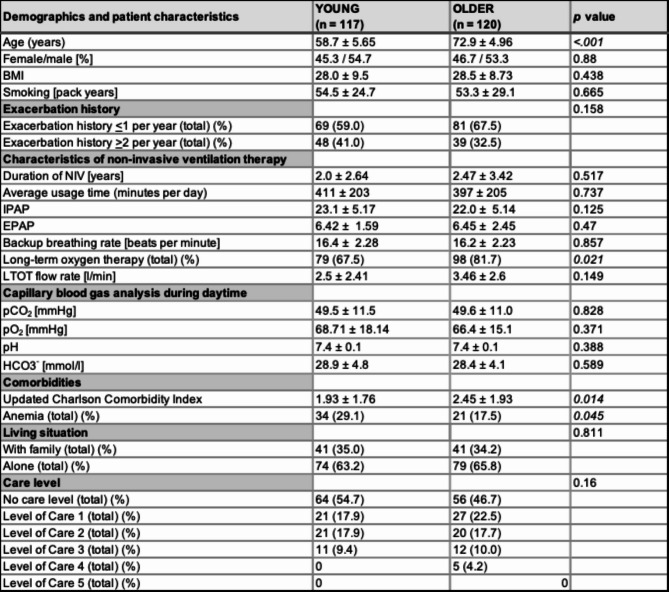
Demographics of the subgroup-analysis young vs. older COPD-patients with type 2 CRF and NIV-therapy. Note: blood gas values were obtained at the time of enrolment of each patient, when long-term NIV therapy was already established. Abbreviation: SD, standard deviation; NIV, non-invasive ventilation, SRI, severe respiratory insufficiency questionnaire

According to the age-differentiated multiple linear regression analyses, only exacerbation frequency and the level of autonomy impairment had a significant negative effect on the SRI Summary Score and subscales in both groups. HRQL was negatively affected by exacerbation frequency in both groups, regardless of age (Young group: -9.2; 95% CI = -14.8/ -3.55 vs. Older group: -6.17; 95% CI = -11.91/ -0.43).

In a stratified analysis, anemia was only associated with a statistically significant reduction in SRI scores in the younger age group (-7.9; 95% CI: -14.0 to -1.75), where anemia was more prevalent.

**Fig. 4:**
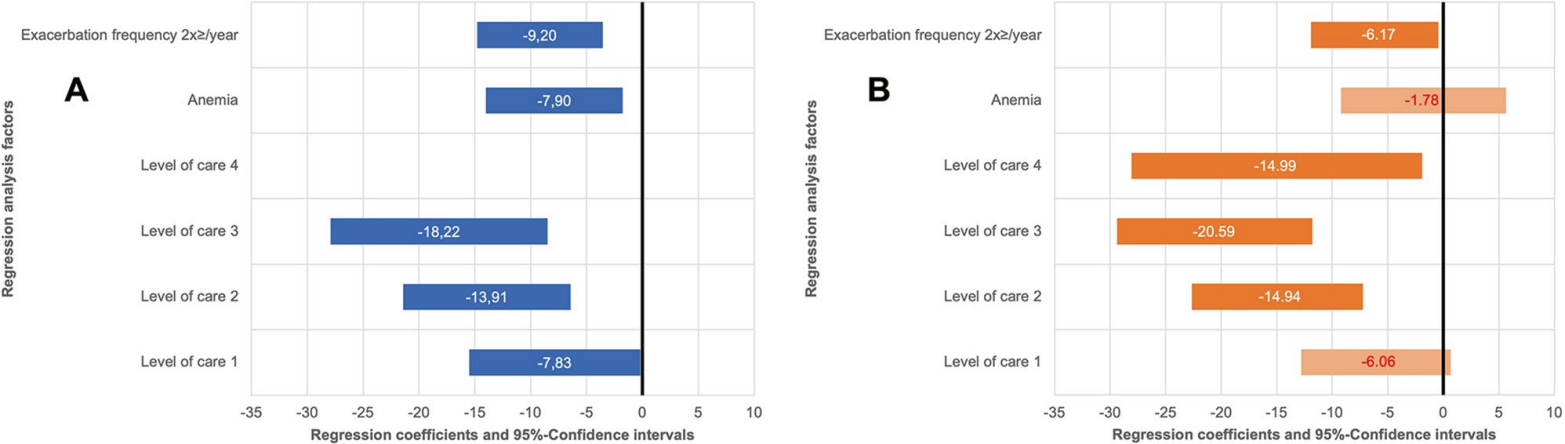
Impact of Clinical Factors on the Severe Respiratory Insufficiency Summary Score in Different Age Groups. Notes: Displayed are the regression coefficients with 95% confidence intervals for factors influencing the Severe Respiratory Insufficiency Summary Score across young (**A**) and older (**B**) groups. Colored bars denote confidence intervals; values represent regression coefficients, with white for statistical significance and red indicating no significant effect. Exacerbation frequency ≥ 2 in the preceding year and anemia defined as hemoglobin < 12 g/dl for females and < 13 g/dl for males per WHO criteria are included. Care levels evaluated by the German Health Insurance Medical Service range from minor (1) to most severe impairments (5), correlating with nursing care complexity

Any level of autonomy impairment had a negative impact on HRQL in younger patients with COPD (level of care 1 (-7.83; 95% CI = -15.5/ -0.18), whereas only higher levels of autonomy impairment (level of care ≥ 2) had a negative impact on the SRI scales in the older age group (level of care 1 (-6.06; 95% CI = -12.81/+0.68). The regression coefficients and their 95% confidence intervals used in the regression analyses for exacerbation history, anemia and level of autonomy impairment are shown in Fig. 4 for the SRI summary score of both groups. Regression coefficients and their 95% confidence intervals for the SRI subscale analysis are displayed in the online data supplement (Additional File 1 and 2).

## Discussion

There is an ongoing debate about the use of long-term home NIV especially in the elderly. Several studies have shown beneficial effects in elderly patients while others found that quality of life only improved in younger patients [3, 36, 37]. Most studies did not differentiate between disease groups. However, the different results regarding HRQL suggest that there may be additional negative or positive factors besides age that influence HRQL in these NIV patients. These factors should be investigated on a disease-specific basis. To the authors’ knowledge, this is the first study to reflect differences in HRQL between younger and older patients with COPD suffering from CHRF receiving long-term home NIV.

The main findings can be summarized as follows: Firstly, it has been shown that there is no difference in HRQL in older patients with COPD compared to younger patients with COPD with CHRF receiving long-term home NIV, despite significantly higher levels of potentially life-shortening comorbidities in older patients. Second, the same predictors, namely higher exacerbation frequency and level of autonomy impairment, were found to have a negative impact on HRQL in both, younger and older patients. Thirdly, in this study population, anemia as a factor was only associated with a significant reduction in SRI scores in the younger group of patients, reflecting HRQL with more patients suffering from anemia in this cohort. Fourth, any level of autonomy impairment had a negative effect on HRQL in younger patients, whereas only higher levels of autonomy impairment negatively influenced older patients HRQL. Fifth, other important clinical parameters, such as BMI, sex, duration of NIV, depression, previous rehabilitation treatment, smoking status, and level of potentially life-shortening comorbidity, which were selected as independent confounders for the regression analysis did not have a negative effect on HRQL.

The presented results contradict some of the cited studies showing no difference in HRQL between younger and older patients with COPD [15, 16]. Moreover, the mean SRI was consistent with earlier studies after initiation of long-term NIV indicating that both age groups benefited within the expected range [5, 6, 33]. This underscores the fact that despite limited resources in healthcare systems the current European and German guidelines generally support the use of long-term NIV to improve health outcomes regardless of age, and highlighting the beneficial effects on HRQL [14, 38, 39].

On the other hand, age related differences in factors influencing HRQL life become more probable when looking at survival data after NIV initiation from a large European cohort and the data of the present study. In the present study age discrepancy between the younger and older patients is high (mean age of 58.7 ± 5.65 years in the younger group versus 72.9 ± 4.96 years in the older group). Looking at the European cohort study only between 25 and 30% of patients with obstructive airways disease survived for more than five years after NIV initiation, however > 60 years of age was associated with a higher mortality [19]. The likelihood of a transition from the younger group to the older group can therefore be considered as small.

Anemia has a negative impact on HRQL, as a previous study has shown in patients with COPD with CHRF receiving NIV [15]. Other studies showing a negative impact of anemia in COPD did not differentiate by patient age [40, 41]. Our study revealed that anemia was associated with lower HRQL in the younger subgroup where its prevalence was higher. This might indicate that these younger patients typically present with a more severe chronic systemic inflammation that disrupts erythropoiesis, thereby leading to anemia, possibly caused by COPD itself, and leading to an earlier death, although further studies are needed here [42, 43].

In contrast, the older group was more affected by potentially life-shortening comorbidities, which supports the higher mortality rate of patients above 60 years of age in the European cohort [18]. However, the uCCI score, which quantifies the burden of these comorbidities, did not significantly influence HRQL. This might indicate a potential disconnection between the severity of comorbidities and how patients perceive their disease-specific quality of life in COPD with CHRF. Despite a potential floor effect in these heavily affected patient cohort, it is remarkable that the probability of mortality within twelve months (resembled by the uCCI) did not affect HRQL significantly in comparison to anemia, level of impairment and exacerbation frequency.

Our study confirmed previous data that exacerbation frequency and autonomy impairment levels were both independent negative factors influencing HRQL in patients with COPD with CHRF receiving NIV. Moreover, the higher the level of autonomy impairment the worse was the impact on HRQL [16], and it should be noted that in the younger group, being in any level of care had a negative effect on HRQL, whereas in the older group this was only observed from level 2 onwards, highlighting the complexity of the topic [15, 16]. However, the present analysis could serve as an argument that patient-specific management strategies could positively influence these factors and prevent further loss of autonomy [44, 45, 46]. Other studies have indicated that an effective management that reduces the frequency and severity of exacerbations can significantly decrease the 1-year mortality and improve HRQL [11, 18, 47, 48, 49].

Based on this, interventions focused on enhancing physical capability and maintaining autonomy might be crucial for improving HRQL. The level of patient’s autonomy, especially in daily activities, emerges as a pivotal factor in determining HRQL [16].

Considering these findings, future research on long-term NIV in patients with COPD should prioritize defining benefits for specific groups acknowledging age-differentiated differences. By integrating this nuanced understanding of COPD with comprehensive care plans that address both medical and functional aspects, patient outcomes and HRQL might be improved.

This study has several limitations. First, the heterogeneity of the cohort - including both inpatients and outpatients - may have introduced variability in clinical status and care settings. Second, although patients were eligible after at least one month of NIV, the majority had been on long-term therapy prior to inclusion (median duration 2.23 years; IQR 0.4–2.8). In a small subset with shorter NIV duration, exacerbation history and HRQL may partially reflect the pre-NIV period. However, as there was no significant association between NIV duration and HRQL, and the proportion of patients affected was small, the overall risk of bias is likely to be low.

However, using the SRI questionnaire patients’ HRQL is assessed for a timespan of two weeks prior to enrolment, a period when all participants were at home. Moreover, this approach was designed to reflect real-life clinical scenarios and included a substantial number of patients with COPD with severe CHRF, focusing exclusively on those with COPD to avoid confounding by other causes of chronic respiratory insufficiency. The observed association between anemia and reduced HRQL in the younger subgroup is based on stratified analysis without formal interaction testing and should be interpreted within the context of the study design, including possible power limitations in the older group. Another limitation is the single-center design of the study, which may not fully capture the diversity of a wider population, particularly regarding the age distribution of participants. In addition, despite the rigorous analysis and review of previous studies, confounding factors may have been overlooked that could have influenced the results of the study.

## Conclusion

The present study has shown that health related quality of life (HRQL) in patients with COPD suffering from chronic hypercapnic respiratory failure receiving long-term home NIV does not differ between younger and older patients. In addition, exacerbation frequency, the level of autonomy impairment and anemia have a major influence on health-related quality of life and age differences should be considered. These factors should therefore be the topic of further investigation and should be considered as confounders when looking at health related quality of life in patients with COPD receiving long-term home NIV.

## Electronic supplementary material

Below is the link to the electronic supplementary material.

Supplementary Material 1: Additional file 1: 95% Confidence Interval for Multiple Regression Analysis of Predictors in young Patients with COPD with Type 2 CRF. Notes: This figure presents the 95% confidence intervals for regression coefficients associated with the Severe Respiratory Insufficiency (SRI) Summary Score in the young group. Each bar illustrates the coefficient's size, with surrounding error bars indicating confidence intervals derived from the coefficient's variance. Coefficients crossing the zero line are statistically non-significant, suggesting an indeterminate impact on the SRI score. Exacerbation frequency is defined as two or more episodes in the year before study inclusion. Anemia conforms to WHO criteria with hemoglobin below 12 g/dl for females and below 13 g/dl for males. The level of care, indicating autonomy impairment severity, is evaluated by the German Health Insurance Medical Service, ranging from minor (level 1) to most severe (level 5) challenges in nursing care. Abbreviations: COPD, chronic obstructive pulmonary disease; CRF, chronic respiratory failure; SRI, Severe Respiratory Insufficiency Questionnaire; SS, Summary Scale; RC, Respiratory Complaints; PF, Physical Functioning; AS, Attendant Symptoms and Sleep; SR, Social Relationships; AX, Anxiety; WB, Psychological Well-Being; SF, Social functioning.

Supplementary Material 2: Additional file 2: 95% Confidence Interval for Multiple Regression Analysis of Predictors in Older Patients with COPD with Type 2 CRF. Notes: This figure presents the 95% confidence intervals for regression coefficients associated with the Severe Respiratory Insufficiency (SRI) Summary Score in the older group. Each bar illustrates the coefficient's size, with surrounding error bars indicating confidence intervals derived from the coefficient's variance. Coefficients crossing the zero line are statistically non-significant, suggesting an indeterminate impact on the SRI score. Exacerbation frequency is defined as two or more episodes in the year before study inclusion. Anemia conforms to WHO criteria with hemoglobin below 12 g/dl for females and below 13 g/dl for males. The level of care, indicating autonomy impairment severity, is evaluated by the German Health Insurance Medical Service, ranging from minor (level 1) to most severe (level 5) challenges in nursing care. Abbreviations: COPD, chronic obstructive pulmonary disease; CRF, chronic respiratory failure; SRI, Severe Respiratory Insufficiency Questionnaire; SS, Summary Scale; RC, Respiratory Complaints; PF, Physical Functioning; AS, Attendant Symptoms and Sleep; SR, Social Relationships; AX, Anxiety; WB, Psychological Well-Being; SF, Social functioning.

## Data Availability

Data is provided within the manuscript or supplementary information files.

## Abbreviations

BMI: Bodymass index
Chi: X^2^-Chi-squared test
CHRF: Chronic hypercapnic respiratory failure
COPD: Chronic obstructive pulmonary disease
CRF: Case report form
Hb: Haemoglobin
HRQL: Health related quality of life
MCID: Minimal clinically important difference
NIV: Non-invasive ventilation
SRI: Severe Respiratory Insufficiency Questionnaire
uCCI: Updated Charlson Comorbidity Index
