# Supplementary figures and images for: Age-differentiated comparison of health-related quality of life and impacting factors in patients with COPD receiving long-term home non-invasive ventilation
Source: BMC Pulm Med. 2025 Jun 7;25:284. doi: 10.1186/s12890-025-03737-3 (PMC12144842; doi:10.1186/s12890-025-03737-3)

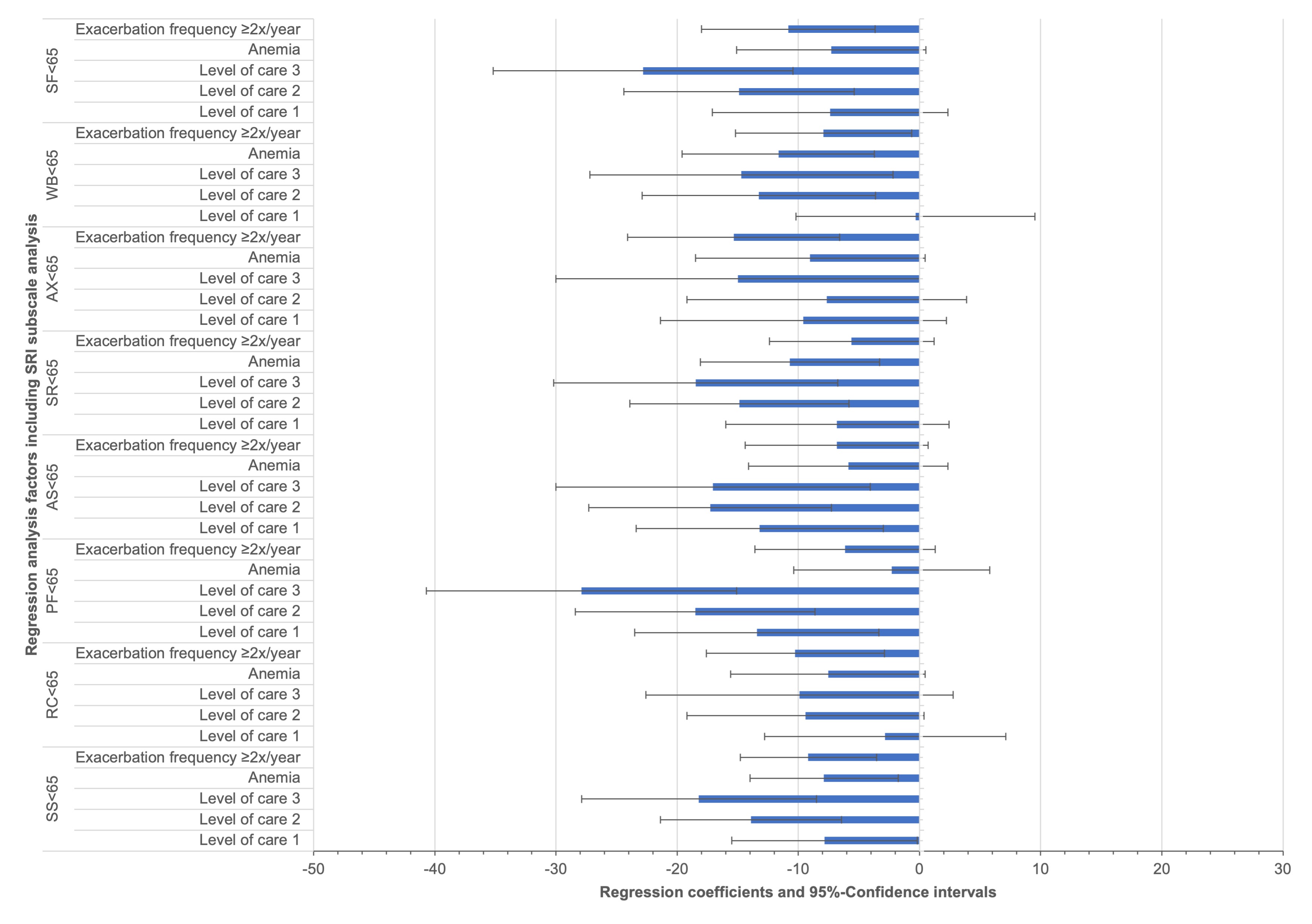

Supplement: Supplementary file 1 — Supplementary Material 1: Additional file 1: 95% Confidence Interval for Multiple Regression Analysis of Predictors in young Patients with COPD with Type 2 CRF. Notes: This figure presents the 95% confidence intervals for regression coefficients associated with the Severe Respiratory Insufficiency (SRI) Summary Score in the young group. Each bar illustrates the coefficient's size, with surrounding error bars indicating confidence intervals derived from the coefficient's variance. Coefficients crossing the zero line are statistically non-significant, suggesting an indeterminate impact on the SRI score. Exacerbation frequency is defined as two or more episodes in the year before study inclusion. Anemia conforms to WHO criteria with hemoglobin below 12 g/dl for females and below 13 g/dl for males. The level of care, indicating autonomy impairment severity, is evaluated by the German Health Insurance Medical Service, ranging from minor (level 1) to most severe (level 5) challenges in nursing care. Abbreviations: COPD, chronic obstructive pulmonary disease; CRF, chronic respiratory failure; SRI, Severe Respiratory Insufficiency Questionnaire; SS, Summary Scale; RC, Respiratory Complaints; PF, Physical Functioning; AS, Attendant Symptoms and Sleep; SR, Social Relationships; AX, Anxiety; WB, Psychological Well-Being; SF, Social functioning. [file 12890_2025_3737_MOESM1_ESM.jpg]

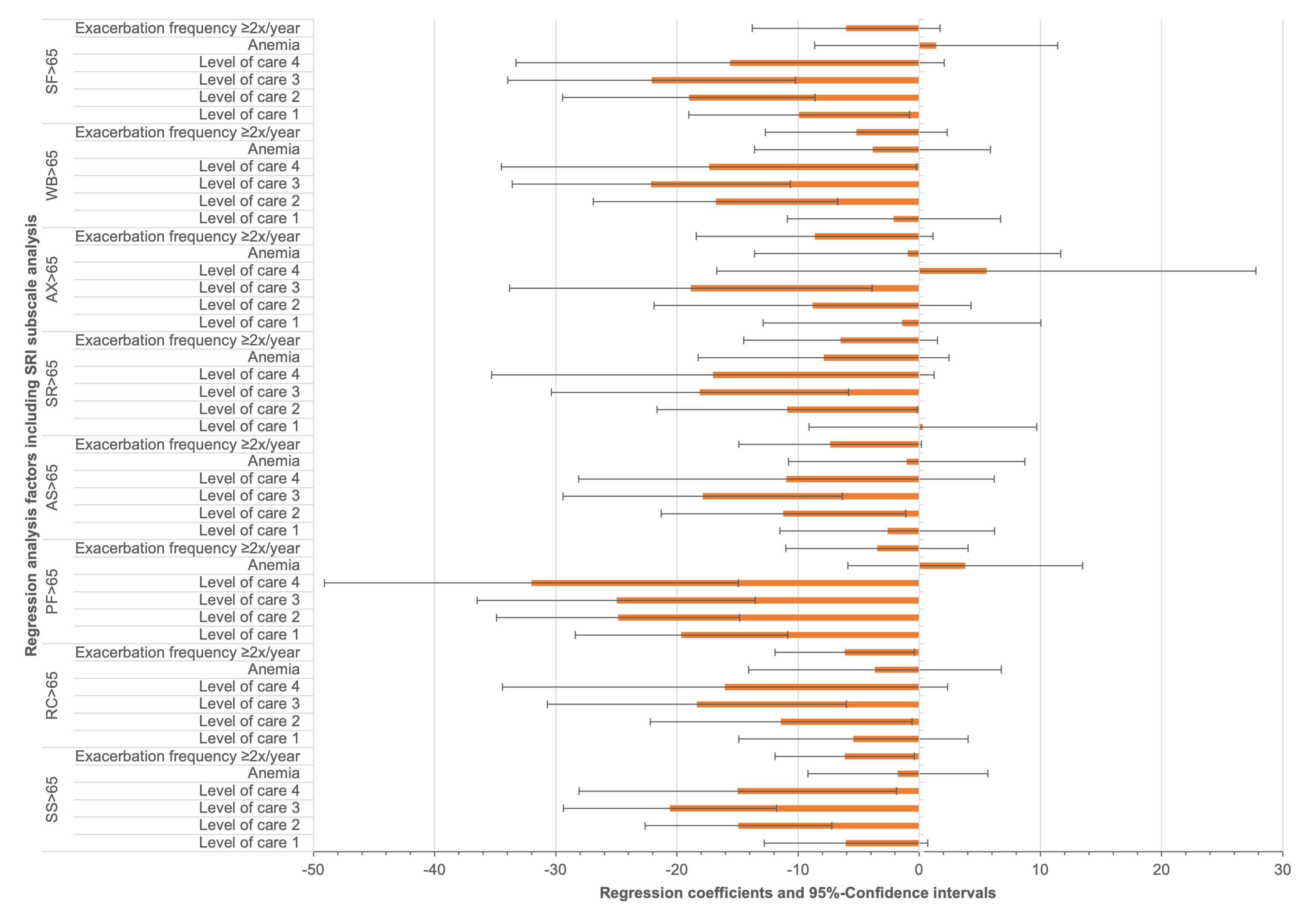

Supplement: Supplementary file 2 — Supplementary Material 2: Additional file 2: 95% Confidence Interval for Multiple Regression Analysis of Predictors in Older Patients with COPD with Type 2 CRF. Notes: This figure presents the 95% confidence intervals for regression coefficients associated with the Severe Respiratory Insufficiency (SRI) Summary Score in the older group. Each bar illustrates the coefficient's size, with surrounding error bars indicating confidence intervals derived from the coefficient's variance. Coefficients crossing the zero line are statistically non-significant, suggesting an indeterminate impact on the SRI score. Exacerbation frequency is defined as two or more episodes in the year before study inclusion. Anemia conforms to WHO criteria with hemoglobin below 12 g/dl for females and below 13 g/dl for males. The level of care, indicating autonomy impairment severity, is evaluated by the German Health Insurance Medical Service, ranging from minor (level 1) to most severe (level 5) challenges in nursing care. Abbreviations: COPD, chronic obstructive pulmonary disease; CRF, chronic respiratory failure; SRI, Severe Respiratory Insufficiency Questionnaire; SS, Summary Scale; RC, Respiratory Complaints; PF, Physical Functioning; AS, Attendant Symptoms and Sleep; SR, Social Relationships; AX, Anxiety; WB, Psychological Well-Being; SF, Social functioning. [file 12890_2025_3737_MOESM2_ESM.jpg]
